# Automatic morphological characterization of nanobubbles with a novel image segmentation method and its application in the study of nanobubble coalescence

**DOI:** 10.3762/bjnano.6.98

**Published:** 2015-04-14

**Authors:** Yuliang Wang, Huimin Wang, Shusheng Bi, Bin Guo

**Affiliations:** 1 School of Mechanical Engineering and Automation, Beihang University, Beijing 100191, P.R. China; 2 Department of Materials Science and Engineering, The Ohio State University, 2041 College Rd., Columbus, OH 43210, USA; 3 School of Material Science and Engineering, Harbin Institute of Technology, Harbin, 150001, P.R. China

**Keywords:** atomic force microscopy, characterization, coalescence, nanobubbles, segmentation

## Abstract

Nanobubbles (NBs) on hydrophobic surfaces in aqueous solvents have shown great potential in numerous applications. In this study, the morphological characterization of NBs in AFM images was carried out with the assistance of a novel image segmentation method. The method combines the classical threshold method and a modified, active contour method to achieve optimized image segmentation. The image segmentation results obtained with the classical threshold method and the proposed, modified method were compared. With the modified method, the diameter, contact angle, and radius of curvature were automatically measured for all NBs in AFM images. The influence of the selection of the threshold value on the segmentation result was discussed. Moreover, the morphological change in the NBs was studied in terms of density, covered area, and volume occurring during coalescence under external disturbance.

## Introduction

Over the last ten years, spherical-capped bubbles on various hydrophobic surfaces in aqueous solvents have gained increasing attention [1–5].These gas bubbles with dimensions of 5–100 nm in height and 100–800 nm in diameter are often referred to as nanobubbles (NBs). The existence of NBs has been verified through various techniques, including atomic force microscopy (AFM) [1,5–9], rapid cryofixation/freeze fracturing [10], neutron reflectometry [11], X-ray reflectivity measurements [12], spectroscopic methods [13], total internal reflection fluorescence excitation [14], and even using an optical visualization approach with a limited resolution [14–15].

NBs have shown their potential in numerous applications. They can be used as vehicles for drug delivery and agents to enhance ultrasound contrast for tumor imaging [16–17]. Studies show that NBs can promote physiological activity of living organisms and increase cell productivity [18]. They are responsible for long-range attractive hydrophobic forces [19–20]. The coalescence of NBs on hydrophobic surfaces is believed to form a gas bridge and leads to long-range attractive forces [19,21]. They are also believed to be the reason for the breakdown of the no-slip boundary condition at the solid–liquid interface on hydrophobic/superhydrophobic surfaces [22–27].

The interaction between NBs and sample surfaces supporting them was also recently investigated. A phenomenon of NB-induced nanoindentions was reported by Wang et al. on an ultrathin polystyrene (PS) film in water [8], and was further confirmed by Janda et al. [28] and Alsawafta et al. [29] on highly ordered, pyrolytic graphite surfaces and gold–gelatin bionanocomposite films, respectively. This raises the possibility that NBs may be used to fabricate nanopatterned surfaces. By investigating the impact of micro/nanostructures on NBs, Wang et al. found that both nanoindentations on a continuously coated PS film and hydrophobic island structures on partially coated PS films can effectively increase their resistance to external disturbancees, which defines NB immobility [9].

The NB properties, and their response to changing experimental parameters, are widely studied. Several studies have shown that increased solution temperature, under ambient conditions are favorable conditions for the generation of NBs [2,30–32]. An increase of the solution temperature results in an increased NB density when the liquid temperature is lower than about 40 °C with an optimal liquid temperature of ≈35–40 °C [2,30–31]. However, when the temperature is higher than 40 °C, the total volume decreases with temperature [32]. Bhushan et al. studied the impact of an electric field applied to the sample substrates of NBs. They found that the NB density and size increased without obvious change to the area covered by the NBs when the substrate bias was increased from 0 to 100 V [33]. NB nucleation is also a function of gas type [34–35]. Among seven different gas types, H_2_, He, CH_4_, N_2_, O_2_, Ar and CO_2_, O_2_-based NBs had the largest diameter and Ar NBs had the largest volume at 25 °C [35]. They also found that the contact angle of the NB was a function of its radius of curvature.

The NB properties, including diameter, height, contact angle, radius of curvature, density and covered area, are normally studied through morphological characterization from AFM images. The first step in NB characterization is image segmentation – a process of identifying the specific areas covered by NBs. With the segmented images, NB-covered area, density, as well as volume can be obtained. Moreover, the cross sections of the NBs can be extracted after image segmentation. With the selected cross sections, the NB diameter and height can be directly measured. By fitting the cross sections as arcs, the NB contact angle and radius of curvature can be obtained [36–39].

The morphological characterization of NBs suffers from several difficulties. First, NB image segmentation is mainly implemented through the threshold method [31,40]. The areas with height larger than the selected threshold value are considered to be NBs. The threshold-based image segmentation method can process hundreds of NBs in one AFM images. However, this method underestimates the NB height, diameter and covered areas. Moreover, the cross sections extracted through the segmentation method represent only a portion of the actual NB cross sections, which lead to the inaccurate estimation of height and diameter. In some studies, the cross sections were manually selected [6,13]. Although the manual selection of cross sections can guarantee accurate NB characterization, only a limited number of NBs can be processed. When hundreds of NBs are involved, an automatic image segmentation method must be employed [2,32–33,35].

In this study, we provide a systematic approach for NB morphological characterization. Here, a novel method was developed to implement automatic image segmentation, which combines the regular threshold method and the active contour method [41] to achieve optimized image segmentation. With this method, the morphological characterization of hundreds of NBs from AFM images was carried out. Moreover, the method was applied to evaluate the morphological changes occurring during coalescence.

## Experimental

### NB imaging

A sample was prepared by spin coating a thin film of PS on a silicon (100) substrate at a speed of 500 rpm. The substrate was cleaned in a sonic bath of acetone and then water. PS particles (molecular weight 350,000, Sigma-Aldrich) were dissolved in toluene (Mallinckrodt Chemical) to a concentration of 0.2 wt % to obtain the solution for spin coating. The contact angle of the PS surface with water was measured to be 95 ± 3° using a sessile drop method.

**Figure 1:**
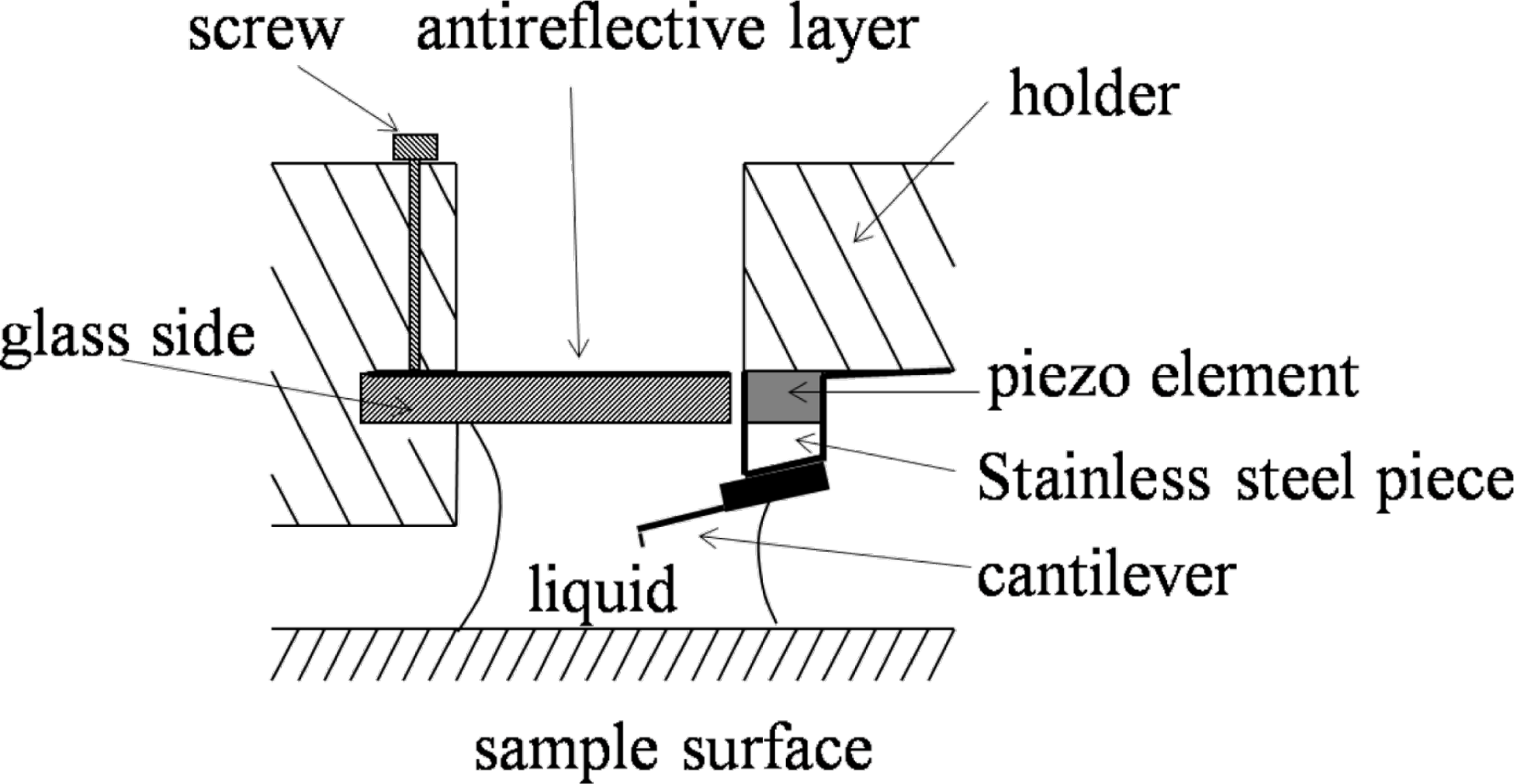
Schematic diagram of the modified tip holder [6].

A commercial AFM (MultiMode III, Digital Instruments) operating in tapping mode was used for imaging the sample. A silicon rotated force-modulated etched silicon probe (RFESP, Bruker Corporation) cantilever with a tip radius of 8 nm and a stiffness of 3 N/m was used. A modified tip holder was used for tapping mode atomic force microscopy (TMAFM) scanning, as was used in our previous studies [6,8–9]. In the general tapping mode operation, the whole liquid cell is excited by a piezoelectric element, which results in a multitude of spurious peaks related to the fluid cell eigenfrequencies. It is difficult to accurately determine the resonance frequency of a cantilever. In this study, a tapping mode tip holder for non-fluid use in air was modified, as shown in Figure 1. A horizontal slot was carved out above the piezo element in the opening of the tip holder to insert a glass slide. When the liquid is added between the glass slide and the substrate, a liquid meniscus is formed between the glass and sample surface for fluid imaging.

The sample was first imaged in air, followed by immersion into deionized (DI) water for imaging by TMAFM. While imaging in water, the drive frequency was chosen to be the resonance frequency. The measured resonance frequency in water was about 25 Hz. The free oscillation amplitude of the cantilever at the working frequency was 7.3 nm. To minimize the force applied to the samples, the setpoint was set at 95% of the free amplitude, which was 6.9 nm. The sample surface was scanned at a rate of 2 Hz and the scan angle was 90°. To study the morphological changes occurring during NB coalescence, higher scanning loads with setpoints of 85% (6.2 nm), 79% (5.7 nm), and 66% (4.8 nm) were applied for a given 2 × 2 μm scanning area. After each high-load scan, the 95% setpoint was selected to check the corresponding changes after coalescence.

### Parameters for NB characterization

In this section, the parameters involved in the morphological characterization of NBs will be individually introduced. For a given AFM image, the total number of NBs can be directly obtained after image segmentation. The NB density is defined as the number of NBs in a unit area. The covered area is the area of the substrate surface covered by NBs. Once the boundaries of the NBs are determined, the area enclosed by the detected NB boundary can be taken as the covered area.

**Figure 2:**
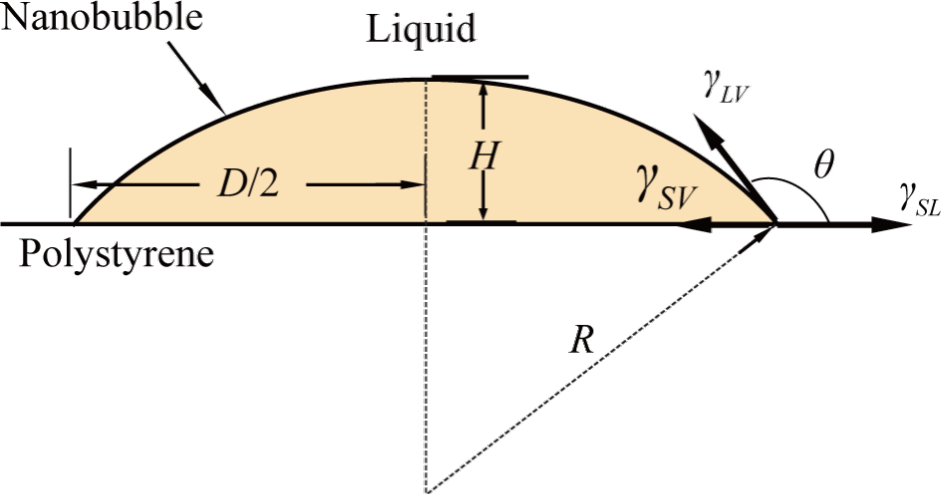
Schematic of a cross section of a NB on a solid PS surface.

Other parameters in NB morphology characterization, such as NB diameter, height, contact angle and radius of curvature, are normally obtained from the cross sections of NBs. A schematic of a cross section of a NB on a PS surface is shown in Figure 2. In the figure, γ_SL_ (72 mN/m for water), γ_SV_, and γ_LV_ are the surface tensions at the solid–liquid, solid–vapor, and liquid–vapor interfaces, respectively. *H*, *D*, *R*, and θ are the NB height, diameter, radius of curvature, and contact angle, respectively. *H* and *D* can be directly obtained from the selected cross section. By assuming the cross section is an elliptical arc, *R* and θ are given as:

[1]
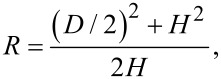


[2]
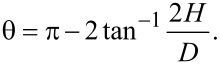


### Algorithms for NB image segmentation

**Figure 3:**
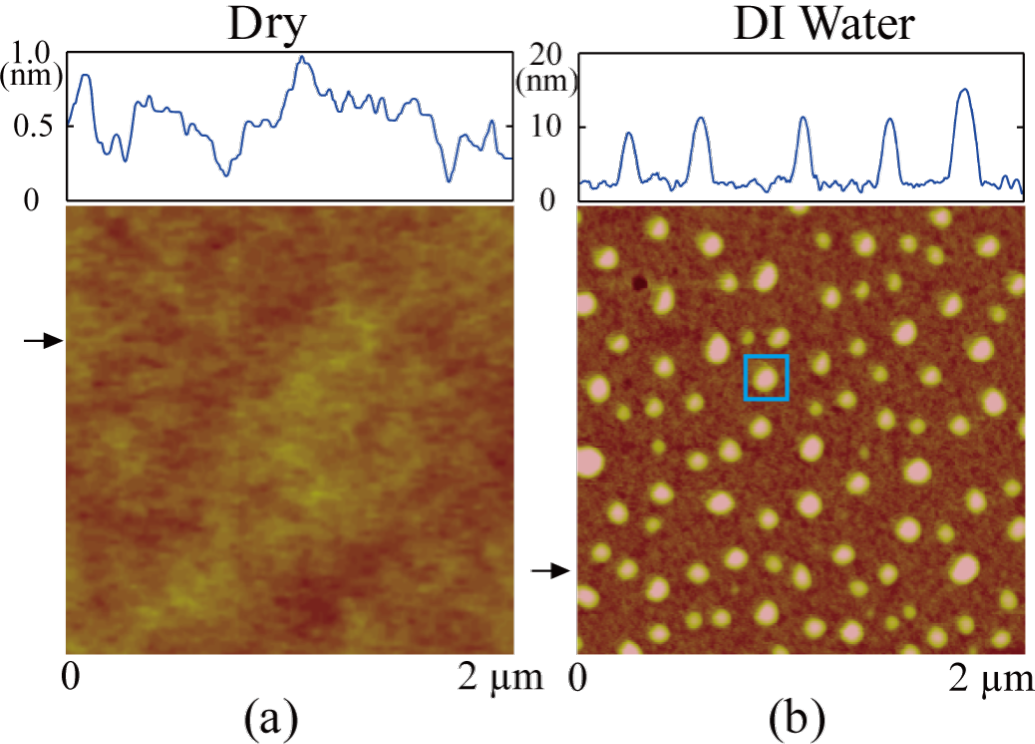
Comparison of AFM images of a PS surface in air (a) and in DI water (b).

In this section, the principles and algorithms for NB image segmentation will be presented step-by-step. The PS surface was first scanned by TMAFM in air and this image is shown in Figure 3a. The root mean square (rms) roughness *R*_rms_ of the image is 0.18 nm. Figure 3b shows the image of the PS surface immersed in DI water. The entire surface is covered with spherical cap-like domains, which are identified as NBs [6]. The *R*_rms_ is 2.8 nm, which is a value much larger than that obtained in air. Due to the mechanical instrumentation drift [42] that occurs during imaging, the obtained AFM images usually need to be flattened. In the regular flattening method, the average height of each scanned line is set to the height of the whole image. As a result, the scan lines containing less NBs have higher height values and appear brighter in AFM images, which results in artifacts in the post-processed images. In this study, the obtained AFM images were flattened by excluding the small areas containing NBs. This operation is a standard function in the AFM operation software and reduces the above mentioned artifacts. This method is referred to as the excluded area flattening method in this study. Figure 3b is a NB image obtained with the excluded flattening method. Figure S1 in Supporting Information File 1 shows the comparison of the AFM image obtained with the linear flattening method and the excluded flattening method.

### Segmentation with the threshold method

**Figure 4:**
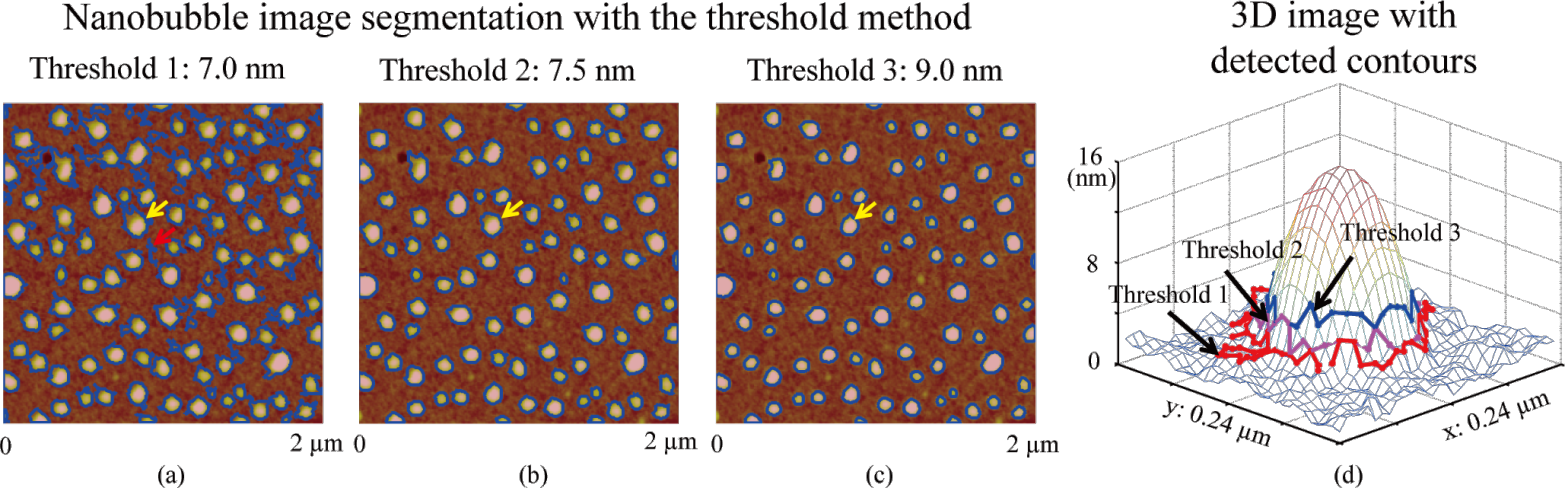
Nanobubble image segmentation using the threshold method with the threshold values of 7.0 nm (a), 7.5 nm (b), and 9.0 nm (c). (d) Mesh plot of a selected NB and the detected contours with the different threshold values. The detected boundaries are strongly related to the selected threshold values.

The image segmentation was first implemented using the threshold method for the image shown in Figure 3b. Figure 4a–c shows the image segmentation results when the threshold values of 7.0 nm, 7.5 nm and 9.0 nm, respectively, were applied. One can see that when the threshold value is set to 7.0 nm, some of the sample substrate was falsely recognized as NBs, as indicated by the red arrow in Figure 4a. This overestimation is called oversegmentation. Therefore, it is clear that the threshold value should be higher than 7.0 nm to avoid oversegmentation. When the threshold value was increased to 7.5 nm, all NBs in the image could be detected, as shown in Figure 4b.

To test the influence of the threshold value on the segmentation result, a higher threshold value of 9 nm was applied, as shown in Figure 4c. A mesh plot of a NB at the location indicated by the yellow arrows in Figure 4a–c is shown in Figure 4d. The detected boundaries with the three different threshold values are also plotted in the mesh plot. As expected, the lowest threshold value (7.0 nm) gives the largest contour and better boundary detection than the higher ones (7.5 and 9.0 nm). This indicates that the threshold method is sensitive to the selection of the threshold value. Moreover, one can see that the method could not achieve optimized boundary detection results even with the lower threshold value: only part of the NB area is enclosed by the detected boundary.

### Optimized NB boundary detection

**Figure 5:**
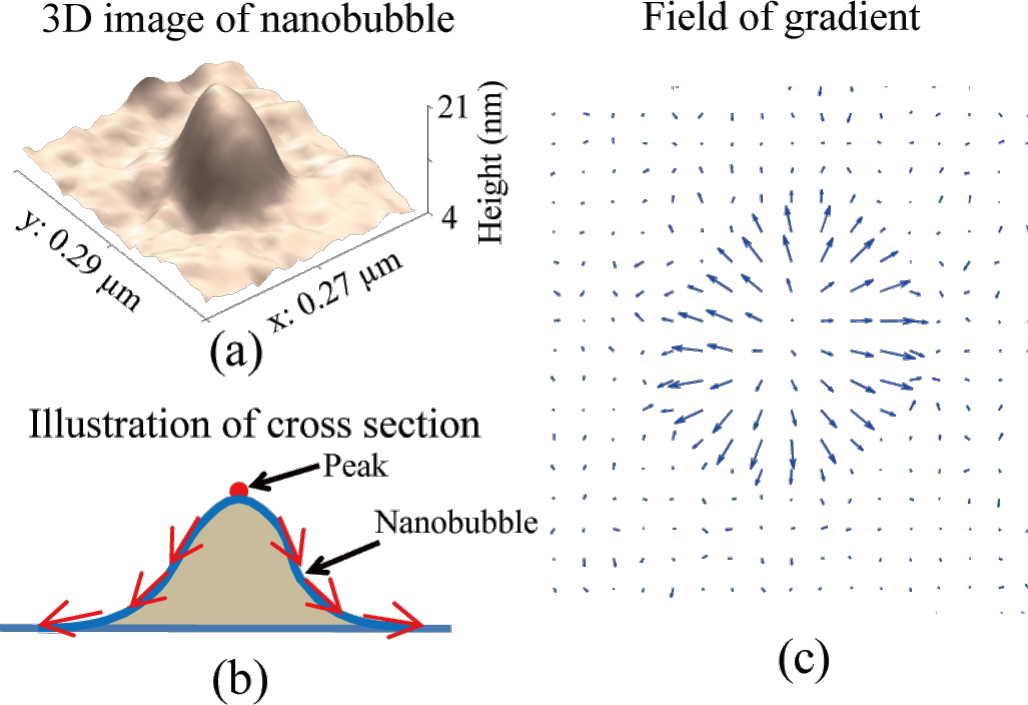
(a) 3D image of a selected NB. (b) Illustration of the NB cross section. (c) Field of gradient of the AFM image.

To obtain an optimized boundary detection, a new approach was proposed in this study. The method utilizes the height distribution information in the AFM images. Figure 5a shows the 3D image of the NB indicated by the yellow arrow in Figure 4a. The apex of NB identifies the NB center, which gradually decreases towards their boundaries, as illustrated in Figure 5b. One can obtain the gradient field of the height by taking the differentiation of the image along both *x* and *y* directions, as shown in Figure 5c. This can be used to define the outline of NB boundaries.

Here, the traditional, active contour method is modified to detect NB boundaries. In the traditional, active contour model, a contour in an image is defined as a parametric contour ν(s) = (*x*(s),*y*(s)) and has an energy function given as [41]:

[3]
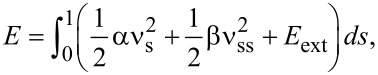


where ν_s_ and ν_ss_ are the first and second order partial derivatives, and α and β are scalar coefficients. The first two terms in Equation 3 are related to the internal energy of the contour, while the *E*_ext_ represents the external energy of the contour. Here, the height of the NBs along the contour is taken as the external energy. The internal energy depends only on the curve geometry and enforces the continuity and smoothness of the curve. The minimization of the total energy *E* satisfies the associated Euler–Lagrange function given as [41]:

[4]
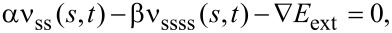


where ν_ssss_ is the fourth order partial derivative of ν(s).

Equation 4 can be numerically solved, as presented by Kass et al. [41] and here we briefly introduce the process. The discrete form of the contour ν(s) can be expressed as a series of points along the contour, given as ν*_i_* = (*x_i_*,*y_i_*) = (*x*(*ih*), *y*(*ih*)), where *h* is the finite step size along the contour. By approximating the derivatives with finite differences, the terms ν_ss_ and ν_ssss_ at point *i* in Equation 4 can then be given as:

[5]
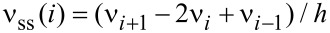


[6]
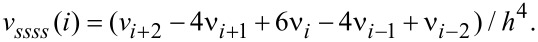


Given *f_x_*(*i*) = ∂*E*_ext_ / ∂*x_i_* and *f_y_*(*i*) = ∂*E*_ext_ / ∂*y_i_*, we have 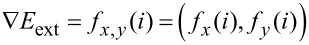.

By combining Equation 4, Equation 5 and Equation 6 and substituting 
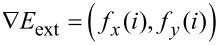
 into Equation 4, the finite difference form of Equation 4 can be given as:

[7]
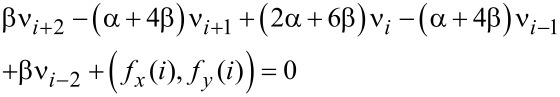


The above finite difference form of the Euler–Lagrange function can be written in matrix notation as

[8]
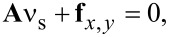


where **A** is a pentadiagonal banded matrix. Equation 8 can be solved through an explicit Euler method between two successive instantaneous time points *t* and *t*−1, given as:

[9]
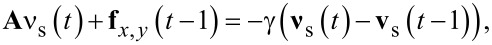


where γ is the step size. Equation 9 can be solved as:

[10]
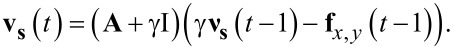


By iteratively solving the Equation 10, the contour will be deformed and converged towards the NB boundary, where the total energy of the contour is minimized. In this study, all calculations were performed with commercial software (MATLAB, USA).

In practice, the original active contour method requires contour initialization, which gives an initial guess of the actual boundary for calculations. In this study, the threshold method and active contour method were combined to carry out automated image segmentation for all NBs in AFM images. Instead of manually drawing the initial contours for individual NBs, we take the contours detected by the threshold method as the initial guess used further in the implementation of the active contour method. The initial contours are mostly located within the actual boundaries and expand outwards.

**Figure 6:**
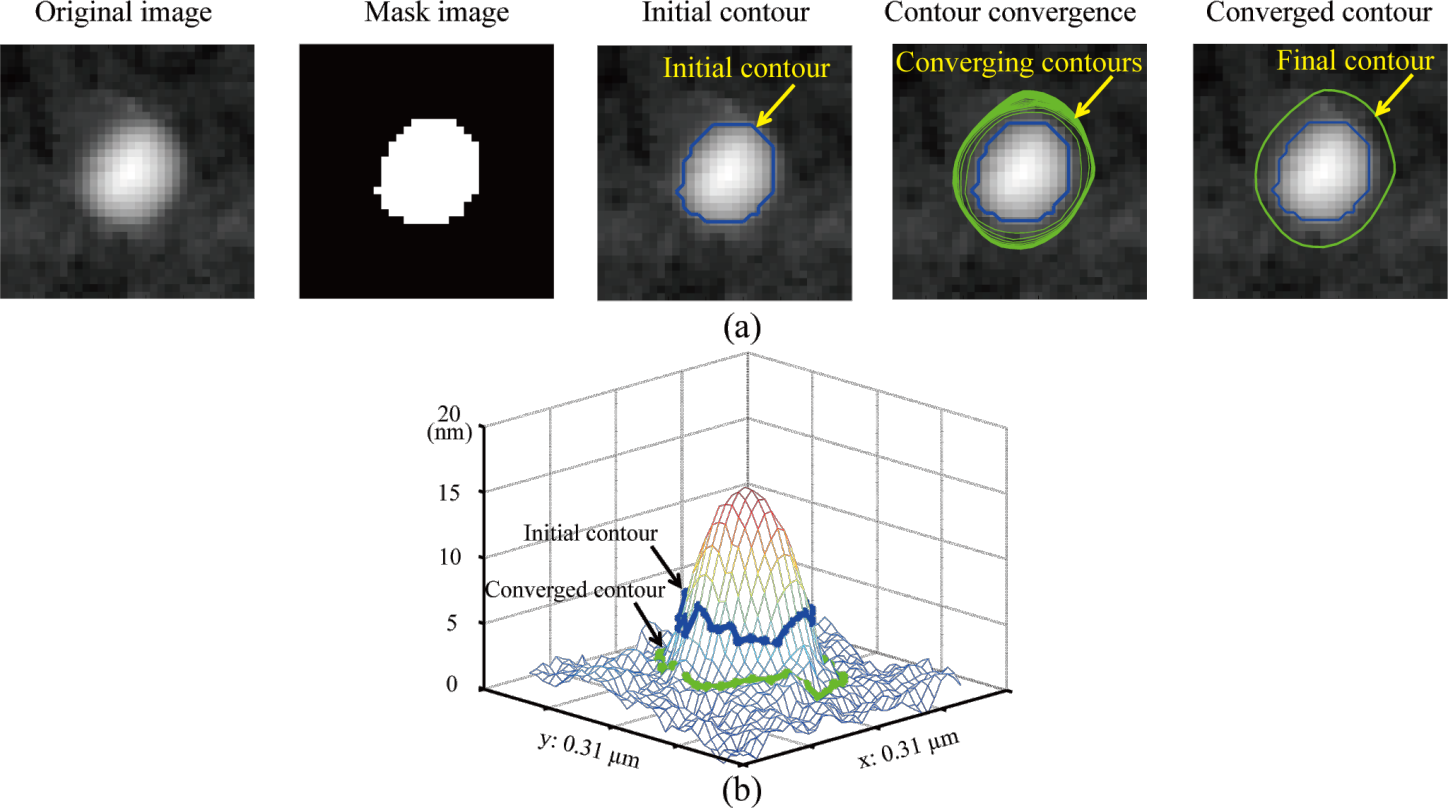
Demonstration of NB image segmentation with the proposed method. (a) The procedure of the NB boundary detection. The first figure shows the raw AFM image. The threshold method is used to select a preliminary mask for the selected NB, as shown in the second figure. The boundary of the mask obtained in the threshold method is extracted and taken as the initial contour (third figure). Driven by the field of gradient of height, the initial contours gradually converge to the boundary, as indicated by the green contours in the fourth figure. The contour is finally converged at the boundary, where the contour achieves the minimum energy (fifth figure). (b) Mesh plot of the NB with the detected boundaries obtained by the threshold method and the proposed method. It is clear that the proposed method provides an optimized boundary detection with a correct detected area as compared with that obtained with the threshold method.

Figure 6a demonstrates the boundary detection for a selected NB using the proposed method. The first image is a raw AFM image of a selected NB. A mask was obtained after applying the threshold method (threshold = 10 nm), as shown in the second image. The boundary of the mask is extracted to serve as the initial contour, as shown in the third image. The area enclosed by the initial contour for this image is 9333 nm^2^. Driven by the field of gradient, the initial contour expands outwards as indicated by the green contours shown in the fourth image. The contour stops at the NB boundary where it achieves the minimum energy, as shown in the fifth image. The area enclosed by the contour is 16714 nm^2^, which is much larger than that obtained with the threshold method. Figure 6b shows a comparison of the contours obtained by the threshold method and the proposed method as a mesh plot of the selected NB. One can see that the contour obtained with the proposed method (green contour) converges to the actual boundary of the NB and provides a much better estimation of the boundary than that obtained with the threshold method (blue contour).

The proposed method can also be used to detect the NB volume. In this study, the average height along detected boundaries is defined as the bottom of NB. The volume enclosed by the NB surface and the horizontal plane determined by the detected bottom is taken as NB volume. The detected volumes for the example given are 3.6 × 10^4^ nm^3^ and 6.3 × 10^4^ nm^3^ from the threshold method and the proposed method, respectively. One can see that the proposed method has a much better estimation of volume than the threshold method.

**Figure 7:**
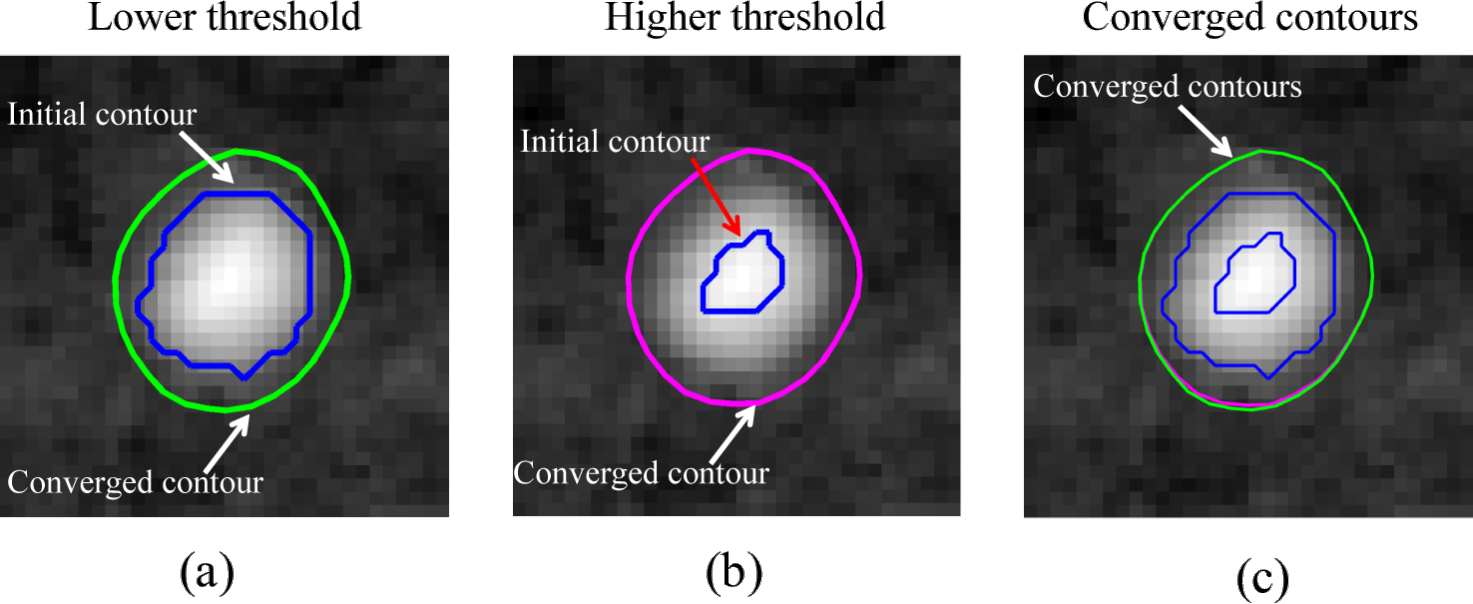
The proposed method is robust to the selection of the threshold value during contour initialization. (a) Initialized contour obtained with a lower threshold value (10 nm) and corresponding converged contour obtained using the proposed method. (b) Initialized contour obtained with a higher threshold value (18 nm) and corresponding converged contour. (c) Comparison of the initialized contours and the converged contours obtained with the different threshold values. The coverged contours are superimposed on each other, which indicates that the proposed method is robust to the selection of the threhold value.

Figure 7 shows the comparison of the contour expansion results with different threshold values during contour initialization. The blue contours in Figure 7a,b are initialized contours with threshold values of 10 nm and 18 nm, respectively. The green contour in Figure 7a and the purple contour in Figure 7b are converged contours using the proposed method with corresponding initial contours. Although the initialized contours are quite different, the converged contours are superimposed on one other, as shown in Figure 7c.

## Results and Discussion

In this section, NB characterization was implemented using the proposed image segmentation method. Additionally, the change in morphology of the NBs during coalescence was studied.

### Image segmentation with the proposed method

**Figure 8:**
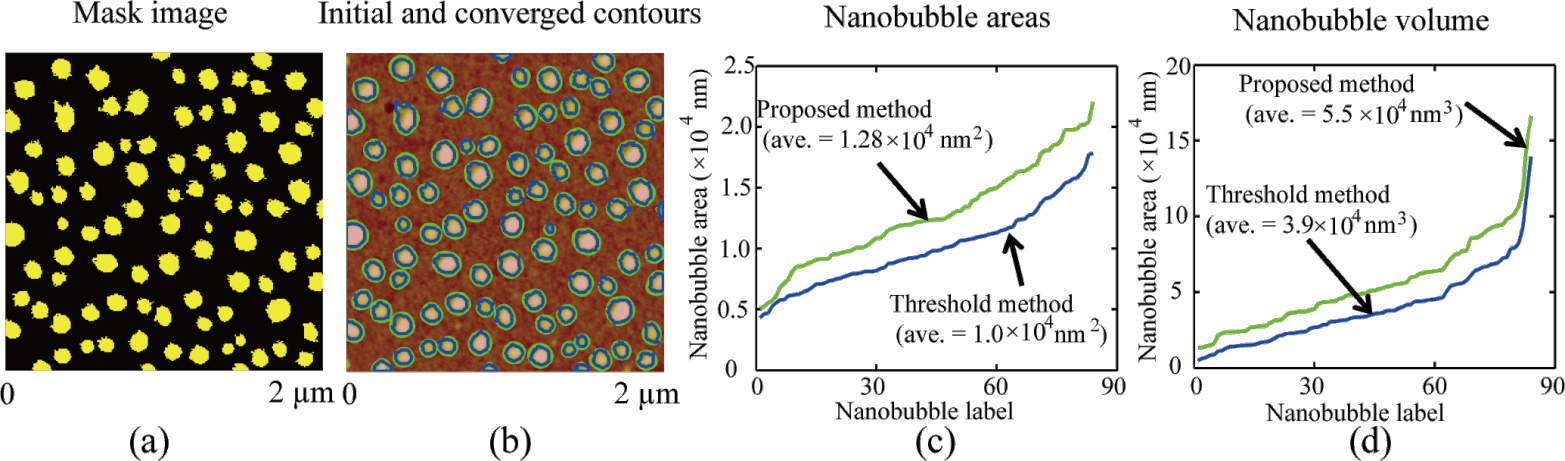
Implementation of image segmentation for all NBs in an AFM image. (a) Mask image obtained using the threshold method. The boundaries of the mask areas were taken as the initial guesses for boundary detection. (b) Initial contours (blue) and converged contours (green) for individual NBs. (c,d) Comparison of covered areas and volumes detected with the threshold method and the proposed method. One can see that both the covered areas and volumes detected with the threshold method are underestimated compared with that obtained by the proposed method.

The AFM image shown in Figure 3b was segmented with the proposed method. First, the threshold method (threshold = 7.5 nm) was applied to the image. A mask image was obtained, as shown in Figure 8a. The boundaries of the masks were extracted and taken as the initial contours for each NB. In Figure 8b, the blue contours are the initialized contours extracted from Figure 8a. With the proposed method, the initial contours converge towards the actual NB boundaries. The green contours in Figure 8b are the converged contours. They clearly enclose larger areas and thus provide a better estimation of the boundaries. Figure 8c shows the comparison of the covered areas enclosed by the contours detected with the threshold method and the proposed method. The NBs were numerically labeled by increasing areas detected by the threshold method. The average value of the covered area detected by the proposed method is 1.28 × 10^4^ nm^2^, which is much larger than that of 1.0 × 10^4^ nm^2^ detected by the threshold method. Figure 8d compares the detected NB volumes for the two different methods. Similarly, the average volume detected by the proposed method is 5.5 × 10^4^ nm^3^, which is much larger than that detected by the threshold method (3.9 × 10^4^ nm^3^).

### Morphological characterization of NBs

The morphological characterization for the NBs found in the AFM image was automatically implemented and the NB boundaries were detected. To obtain the height information, the contact angle and radius of curvature, as well as the cross sections for individual NBs were first extracted, whereby the NB centroids must first be determined. In this study, the centroid (*x*_c_, *y*_c_) of a NB was calculated within the detected boundary with the following equation:

[11]
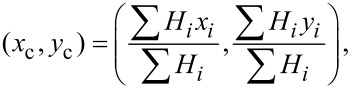


where *H_i_* is the height of the *i*th point within the detected NB boundary and (*x_i_*, *y_i_*) is the coordinate of the point in the image. To obtain the cross section, a line is automatically drawn along the fast scan direction in the NB image across the detected centroid. The two intersection points of the line with the detected boundary result. The portion of the profile between the two interaction points is selected as the cross section of the NB. With the detected cross sections, the NB height, width, contact angle and radius of curvature can be obtained.

**Figure 9:**
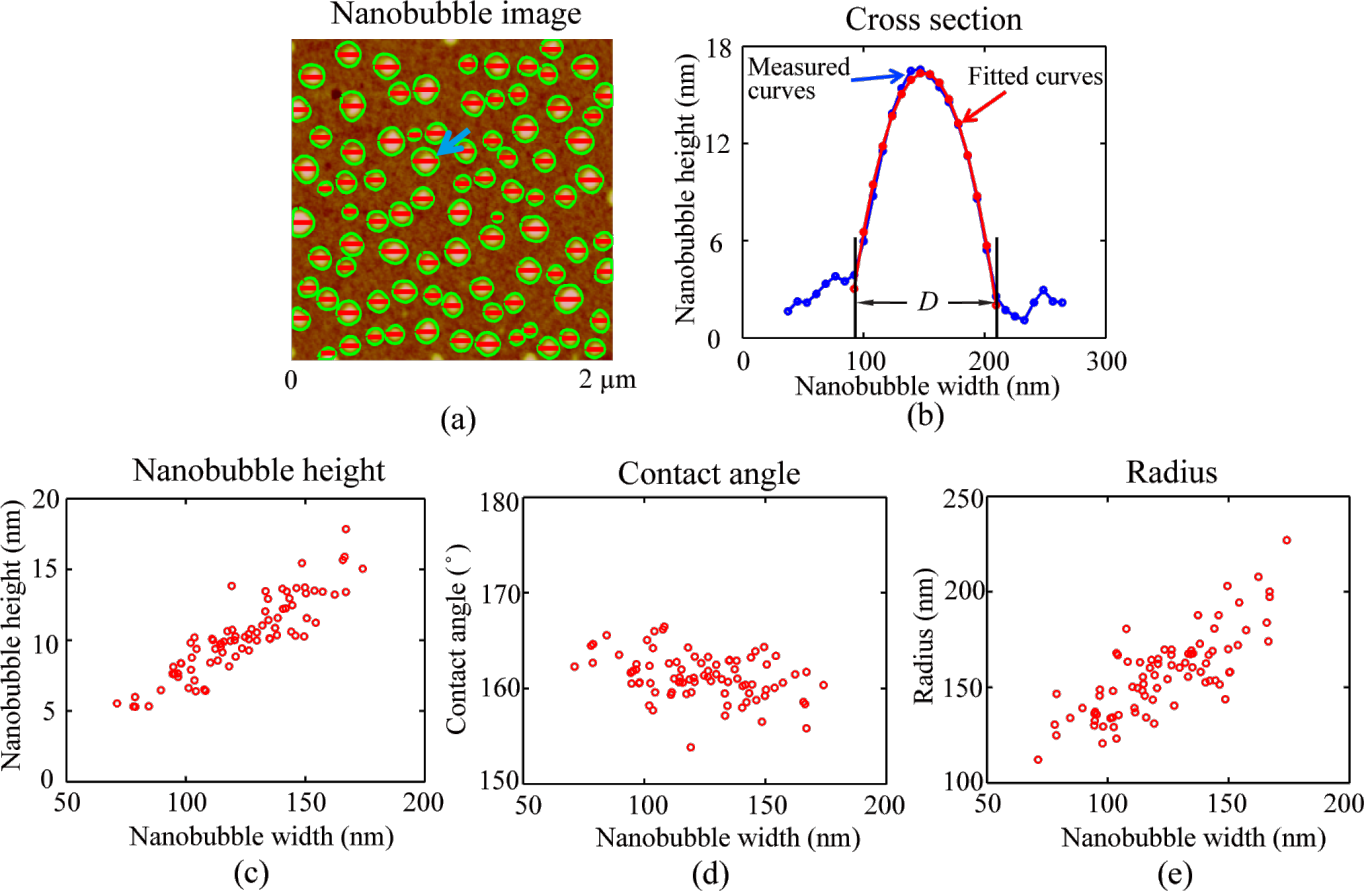
Morphological characterization of NBs detected with the proposed method. (a) Automated extraction of the NB cross sections after image segmentation. (b) Cross section of the NB indicated by the blue arrow in (a) and a corresponding least squares fit curve by fitting the profile as a circular arc. (c–e) The height, contact angle, and radius of curvature as a function of width for all detected NBs in the AFM image, respectively. The NB height and radius of curvature increase with width, while the contact angle decreases with width.

Figure 9a shows the detected boundaries along with the automatically selected cross sections for all NBs in the AFM image. The blue curve in Figure 9b shows the section profile across a NB, indicated by the blue arrow in Figure 9a. The cross section was automatically selected as previously described. Through the cross section, the NB width *D* can be directly obtained. The selected cross section was then fitted as an elliptical arc using the least squares fitting method, as shown by the red curve.

Figure 9c–e shows the height, contact angle, and radius of curvature as a function of width, respectively, for all NBs in Figure 9a. One can see that the NB height increases with increasing width. The NB contact angle varies in between 150° and 170° and slightly decreases with increasing width. The measured contact angle and the correlation between contact angle and NB size is consistent with that reported elsewhere [35,43–44]. In their study, they claimed that the contact angle is a function of radius of curvature. This is mainly due to the existence of line tension along the three phase contact line. Here one should note that the tip radius, contact angle, as well as width shown in Figure 9 are the statistical values directly obtained from AFM images. It is known that the AFM images are a combination of sample topography and the shape of the cantilever tip [45–46]. Here we take the radius of curvature as an example. For the tip used in this study, the half cone angle, α_tip_, is less than 20°. Since contact angle θ for NB imaging is much larger than (90° + α_tip_), one can assume the NBs are probed only at the spherical tip apex and the side wall of the tip does not touch the NBs. The measured radius of curvature, *R*′, is given as *R*′ = *R* + *R*_tip_, where *R*_tip_ (8 nm in this case) is the radius of curvature of the AFM tip [46]. One can see that tip convolution leads to an overestimation of the radius of curvature. Assuming the NB heights are not influenced by the tip shape, the NB width and contact angle can then be obtained.

**Figure 10:**
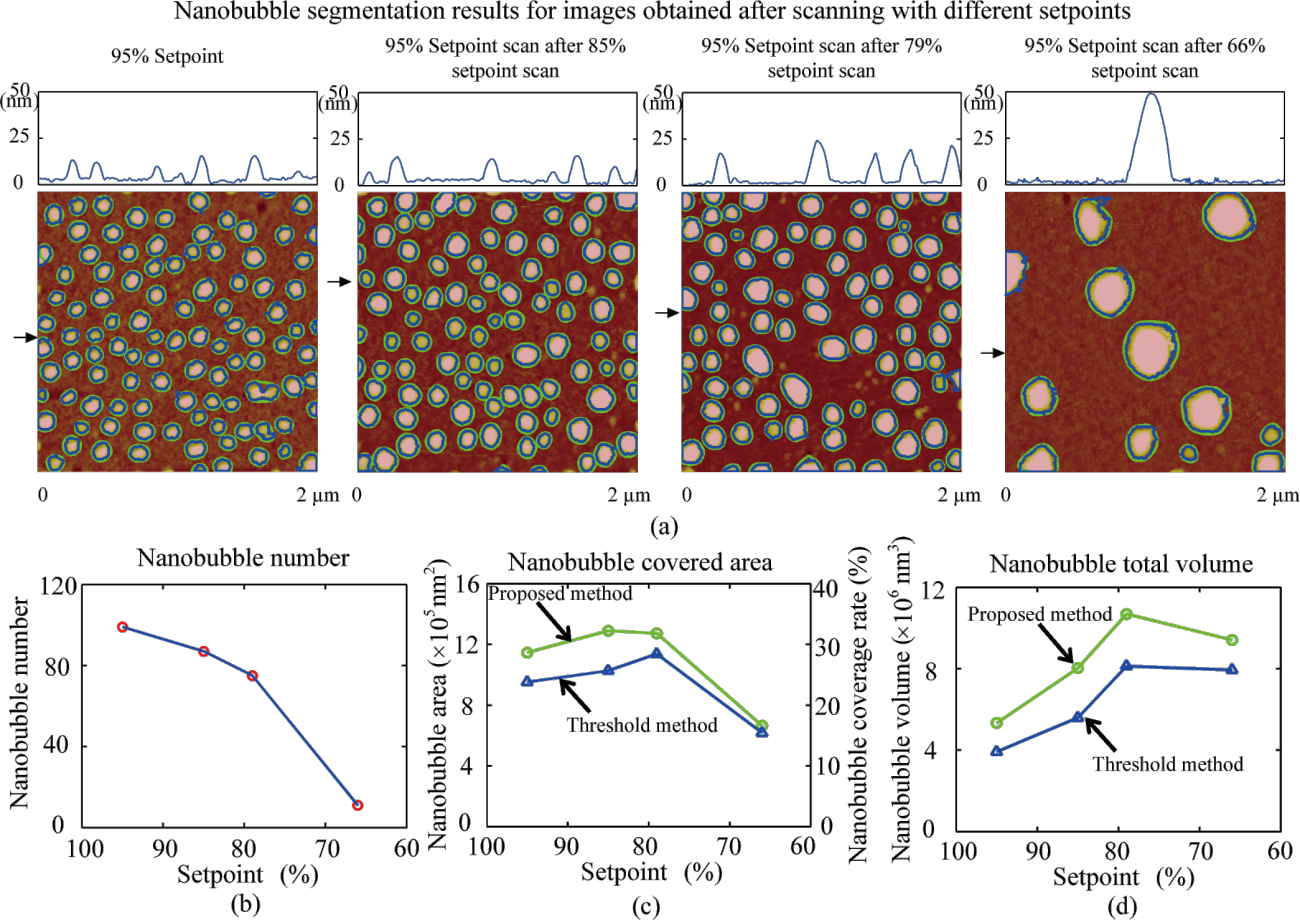
Change in the number of NBs, covered area, and total volume in the same scanning area as a function of decreased setpoint (increased scan load). (a) AFM image and corresponding segmentation results for a series of AFM images scanned with different setpoints. The first figure is the image scanned with a 95% setpoint. The second, third, and fourth images are the obtained with a 95% setpoint preceded by scanning with 85%, 79%, and 66% setpoints, respectively. With decreasing a setpoint, NB coalescence occurred, resulting in a decreased number of NBs and increased NB size. (b–d) number of NBs, covered area, and total volume in the same scan area as a function of applied setpoint. The number of NBs decreases with decreasing setpoint. The total covered area and total volume first increased and then decreased with the setpoint value.

The proposed method was used to study morphological changes in NBs in terms of number, covered area, and volume during coalescence. In this study, a same sample area was imaged with different setpoints and all of the obtained images were postprocessed with the flattening method. For the area, a high setpoint (95%) was first applied to obtain the initial image, as shown in the first image of Figure 10a. After that, a lower setpoint of 85% was applied and NB coalescence occurred [6]. This was confirmed with another high setpoint (95%), as shown in the second figure of Figure 10a. Similarly, setpoints of 79% and 66% were applied to scan the area. After each high-load (lower setpoint) scan, the 95% setpoint scanning was selected to check the corresponding changes after coalescence. The third and fourth images in Figure 10a show images after further NB coalescence. Apparently, the density of NBs decreases with increased scan load.

The AFM images shown in Figure 10a were processed with the proposed image segmentation method. The blue contours in the figures are initial contours extracted using the threshold method and the green contours are detected boundaries with the proposed method. For each image, the threshold value was carefully selected. First, the threshold value should be low enough to cover as large of an area as possible. Second, the selected threshold value should not cause oversegmentation. For the four images shown in Figure 10a, the threshold values of 8.0 nm, 7.5 nm, 7.5 nm, and 7.6 nm were selected. In the images, the blue contours are the initial contours obtained with the threshold method, while the green contours are the detected boundaries with the proposed method. Figure 10b shows the number of NBs as a function of applied setpoint. One can see that the number of NBs first slightly decreased with increased scan load when the setpoint decreased from 95% to 79%. Then, it rapidly decreased when the setpoint decreased from 79% to 66%.

Figure 10c shows the covered area and coverage rate as a function of applied setpoint. Compared with the proposed method, the threshold method underestimated the covered area by about 14%. More importantly, we found the covered area did not monotonically change with decreased setpoint value. The covered area first increases with increased setpoint value when the setpoint is decreased from 95% to 85%. The maximum coverage rate is 32.2%, which was achieved after the 58% setpoint was achieved. Then, the coverage rate decreases from 31.8% to 16.6% when the setpoint is further decreased from 79% to 66%.

In addition to the covered area, the change of the total NB volume in the scan area was studied. The total volume as a function of applied setpoint obtained with the threshold method and the proposed method is shown in Figure 10d. Compared with the proposed method, the threshold method underestimated the volume detection by about 24%. One can see that the total volume first increases when the setpoint is decreased from 95% to 79% and then remains at about 1.0 × 10^7^ nm^3^ for setpoint values between 79% and 66%.

One explanation for the increased total volume could be the deceased inner pressure with increasing size. According to the Laplace–Young equation, the pressure difference ∆*p* across a NB can be given as [47]:

[12]
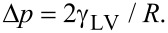


**Figure 11:**
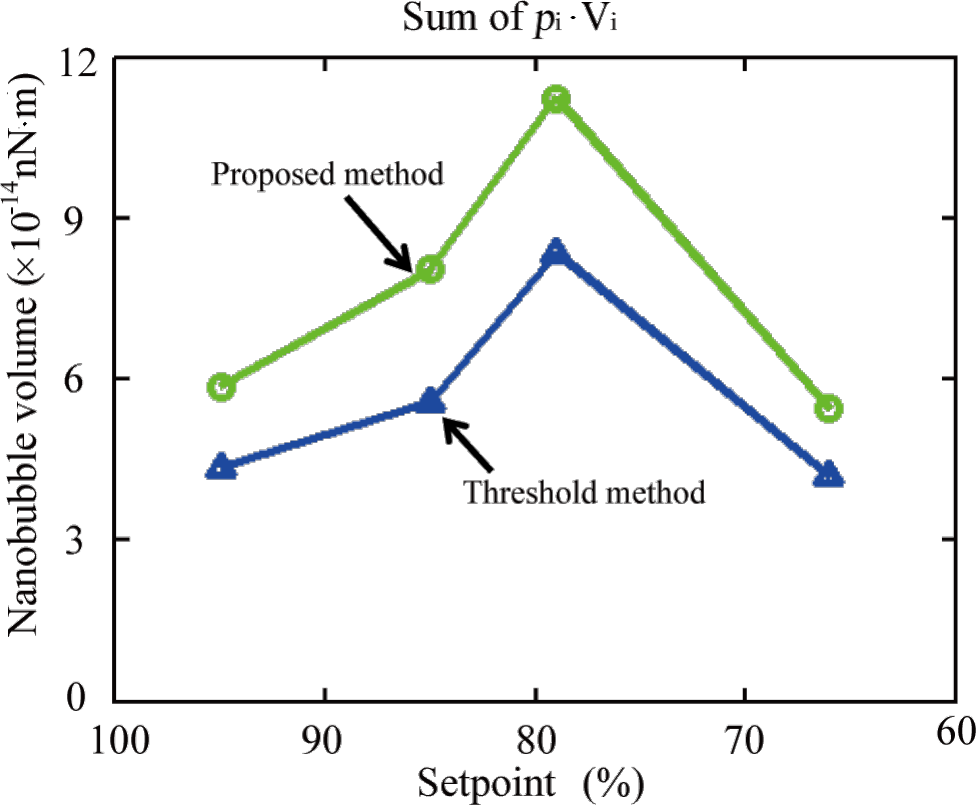
Sum of *p_i_*∙*V_i_* obtained with the threshold method and the proposed method for images shown in Figure 10a.

The inner pressure *p* can be given as:

[13]
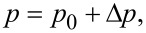


where *p*_0_ is the ambient pressure. With a decreasing setpoint, NB coalescence occurred and the NB size (*R*) increases, which leads to decreased inner pressure, assuming the ambient pressure *p*_0_ is constant during the experiment. The increased NB size will lead to decreased inner pressure. The quantity of gas molecules can be evaluated with *p*∙*V*, where *V* is the NB volume. The decreased pressure will lead to an increased NB volume. In this study, the inner pressure can be obtained by the radius of curvature for each NB using Equation 13. The volume can be directly measured with the detected boundaries. The sum of *p_i_*∙*V_i_* can then be obtained for NBs in each image shown in Figure 10a**.** The result is shown in Figure 11. From this result, one can see that the *p*∙*V* increases with a decreasing setpoint when the setpoint is decreased from 95% to 79%. After that, *p*∙*V* rapidly decreases with decreasing setpoint when the setpoint is decreased from 79% to 66%.

The measured results indicate that the total number of gas molecules trapped in the NBs may not be constant during coalescence. The observation is consistent with that recently reported by Li et al. [48] In their study, they found that the total number of gas molecules in a newly formed NB after coalescence was 112.5% higher than that in the corresponding NBs before coalescence. They stated that the increased number of gas molecules after coalescence chould be due to the existence of an interfacial gas enrichment layer [11,49] and the dynamic equilibrium mechanism between the influx and the outflux around the three phase contact line of the NBs [50]. The inner pressure decreases with increasing NB size, which increases the gas influx into the newly formed NBs. This results in an increased number of gas molecules. However, the reason for the decreased number of gas molecules when the setpoint is decreased from 79% to 66% is still unknown. For the above analysis, the NBs are assumed to be moved and merged into other NBs during coalescence. However, it is still not clear if some NBs were broken during this process, especially when higher loads were applied. One explanation for the decreased *p*∙*V* when the setpoint was decreased from 79% to 66% could be the dissolution of some NBs during coalescence.

## Conclusion

In this study, the morphological characterization of NBs was implemented. Here, a new method was developed for image segmentation through the combination of the threshold method and the active contour method. The threshold method was used to locate the NBs and to obtain their preliminary boundaries. In the active contour method, driven by the gradient of the height, the preliminarily obtained boundaries converge towards actual boundaries and achieve the optimized boundary detection. With the proposed image segmentation method, the diameter, contact angle and radius of curvature for all NBs in AFM images were automatically measured. The results showed that the NB height and radius of curvature increase with its width, while contact angle decreases with increasing width.

The morphological changes in the NBs occurring during coalescence were quantitatively characterized for the first time with the proposed method. A series of scans with setpoints of 95%, 85%, 79%, and 66% were applied to a same scan area. The changes in the NB density, covered area, and volume were quantitatively studied. The results showed that the NB density first gradually decreases when the setpoint was decreased from 95% to 79% and then rapidly decreased when the setpoint was decreased from 79% to 66%. The covered area first increased when the setpoint decreased from 95% to 85%. The maximum coverage rate of 32.2% was achieved at an 85% setpoint value. Then, the coverage rate decreased from 31.8% to 16.6% when the setpoint was decreased from 79% to 66%. The total volume first increased when the setpoint was decreased from 95% to 79% and then stayed at about 1.0 × 10^7^ nm^3^ between the 79% and 66% setpoint values.

## Supporting Information

File 1 Additional experimental information.

## References

[1] Lou S-T, Ouyang Z-Q, Zhang Y, Li X-J, Hu J, Li M-Q, Yang F-J (2000). J Vac Sci Technol, B.

[2] Yang S, Dammer S M, Bremond N, Zandvliet H J W, Kooij E S, Lohse D (2007). Langmuir.

[3] Tyrrell J W G, Attard P (2001). Phys Rev Lett.

[4] Agrawal A, Park J, Ryu D Y, Hammond P T, Russell T P, McKinley G H (2005). Nano Lett.

[5] Holmberg M, Kühle A, Garnæs J, Mørch K A, Boisen A (2003). Langmuir.

[6] Bhushan B, Wang Y, Maali A (2008). J Phys: Condens Matter.

[7] Tyrrell J W G, Attard P (2002). Langmuir.

[8] Wang Y, Bhushan B, Zhao X (2009). Nanotechnology.

[9] Wang Y, Bhushan B, Zhao X (2009). Langmuir.

[10] Switkes M, Ruberti J W (2004). Appl Phys Lett.

[11] Steitz R, Gutberlet T, Hauss T, Klösgen B, Krastev R, Schemmel S, Simonsen A C, Findenegg G H (2003). Langmuir.

[12] Poynor A, Hong L, Robinson I K, Granick S, Zhang Z, Fenter P A (2006). Phys Rev Lett.

[13] Zhang X H, Quinn A, Ducker W A (2008). Langmuir.

[14] Chan C U, Ohl C-D (2012). Phys Rev Lett.

[15] Karpitschka S, Dietrich E, Seddon J R T, Zandvliet H J W, Lohse D, Riegler H (2012). Phys Rev Lett.

[16] Xing Z, Wang J, Ke H, Zhao B, Yue X, Dai Z, Liu J (2010). Nanotechnology.

[17] Yin T, Wang P, Zheng R, Zheng B, Cheng D, Zhang X, Shuai X (2012). Int J Nanomed.

[18] Weber J, Agblevor F A (2005). Process Biochem.

[19] Ishida N, Sakamoto M, Miyahara M, Higashitani K (2002). J Colloid Interface Sci.

[20] Palmer L A, Cookson D, Lamb R N (2011). Langmuir.

[21] Hampton M A, Nguyen A V (2010). Adv Colloid Interface Sci.

[22] Joseph P, Cottin-Bizonne C, Benoît J M, Ybert C, Journet C, Tabeling P, Bocquet L (2006). Phys Rev Lett.

[23] Lauga E, Stone H A (2003). J Fluid Mech.

[24] Ou J, Perot B, Rothstein J P (2004). Phys Fluids.

[25] Sbragaglia M, Prosperetti A (2007). J Fluid Mech.

[26] Wang Y, Bhushan B, Maali A (2009). J Vac Sci Technol, A.

[27] Bhushan B, Wang Y, Maali A (2009). Langmuir.

[28] Janda P, Frank O, Bastl Z, Klementová M, Tarábkova H, Kavan L (2010). Nanotechnology.

[29] Alsawafta M, Badilescu S, Truong V-V, Packirisamy M (2012). Nanotechnology.

[30] Zhang X H, Zhang X D, Lou S T, Zhang Z X, Sun J L, Hu J (2004). Langmuir.

[31] Guan M, Guo W, Gao L, Tang Y, Hu J, Dong Y (2012). ChemPhysChem.

[32] Berkelaar R P, Seddon J R T, Zandvliet H J W, Lohse D (2012). ChemPhysChem.

[33] Bhushan B, Pan Y, Daniels S (2013). J Colloid Interface Sci.

[34] Belova V, Krasowska M, Wang D, Ralston J, Shchukin D G, Möhwald H (2013). Chem Sci.

[35] van Limbeek M A J, Seddon J R T (2011). Langmuir.

[36] Zhang X, Uddin M H, Yang H, Toikka G, Ducker W, Maeda N (2012). Langmuir.

[37] Lüderitz L A C, von Klitzing R (2012). Langmuir.

[38] Zhang X, Chan D Y C, Wang D, Maeda N (2013). Langmuir.

[39] Walczyk W, Schön P M, Schönherr H (2013). J Phys: Condens Matter.

[40] Guo W, Shan H, Guan M, Gao L, Liu M, Dong Y (2012). Surf Sci.

[41] Kass M, Witkin A, Terzopoulos D (1988). Int J Comput Vision.

[42] Wang Y, Wang H, Bi S (2014). AIP Adv.

[43] Song B, Walczyk W, Schönherr H (2011). Langmuir.

[44] Yang J, Duan J, Fornasiero D, Ralston J (2003). J Phys Chem B.

[45] Kameda N, Sogoshi N, Nakabayashi S (2008). Surf Sci.

[46] Borkent B M, de Beer S, Mugele F, Lohse D (2010). Langmuir.

[47] Israelachvili J (1992). Intermolecular & Surface Forces.

[48] Li D, Jing D, Pan Y, Wang W, Zhao X (2014). Langmuir.

[49] Lu Y-H, Yang C-W, Hwang I-S (2012). Langmuir.

[50] Brenner M P, Lohse D (2008). Phys Rev Lett.

