# Supplementary material for: Automatic morphological characterization of nanobubbles with a novel image segmentation method and its application in the study of nanobubble coalescence
Source: Beilstein J Nanotechnol. 2015 Apr 14;6:952–63. doi: 10.3762/bjnano.6.98 (PMC4419579; doi:10.3762/bjnano.6.98)
Supplement: File 1 — Additional experimental information. [file Beilstein_J_Nanotechnol-06-952-s001.pdf]

Supporting Information

for

Automatic morphological characterization of nanobubbles with a novel image segmentation method and its application in the study of nanobubble coalescence

Yuliang Wang[1*] , Huimin Wang[2], Shusheng Bi[1] and Bin Guo[3]

Address: [1]School of Mechanical Engineering and Automation, Beihang University, Beijing 100191, P.R. China, [2]Department of Materials Science and Engineering, The Ohio State University, 2041 College Rd., Columbus, OH 43210, USA and [3]School of Material Science and Engineering, Harbin Institute of Technology, Harbin, 150001, P.R. China

Email: Yuliang Wang* - wangyuliang@buaa.edu.cn

[*]Corresponding author

Additional experimental information

Due to the existence of mechanical drift during imaging, the obtained AFM images usually need to be flattened. In the regular flattening method, the average height of each scanned line is set to the height of the whole image. As a result, the scan lines containing fewer nanobubbles have higher height values and appear brighter in the AFM images after flattening, which results in artifacts in the postprocessed images. In this study, the obtained AFM images were flattened by excluding the areas containing NBs, which is referred to as the excluded area flattening method. This is a standard function in the AFM operation software and could reduce the above mentioned artifacts. This document presents the comparison of AFM images and corresponding image segmentation results associated with the two different flattening methods: regular flattening method and excluded area flattening method.

Figure S1 shows the comparison of an NB image obtained with the two different methods. One can see that the image obtained with the regular flattening method has artifacts, as indicated by the arrow in Figure S1(a). Figure S1(b) has consistent background and clear NB boundaries.

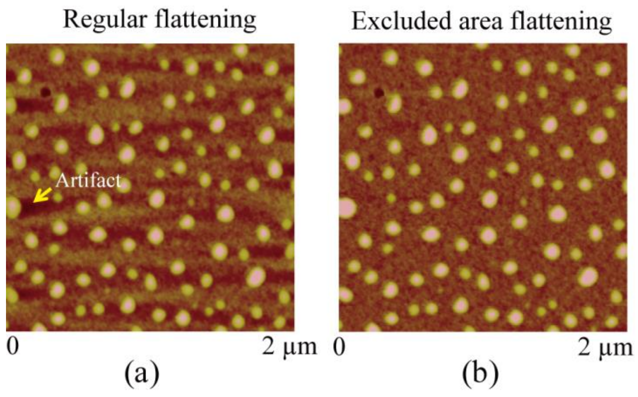

Figure S1: Comparison of an AFM image obtained with the regular flattening method (a) and the excluded area flattening method (b).

In the morphology characterization of nanobubble coalescence, the AFM images obtained with the four different setpoint values and postprocessed by the regular flattening method are shown in Figure S2(a).

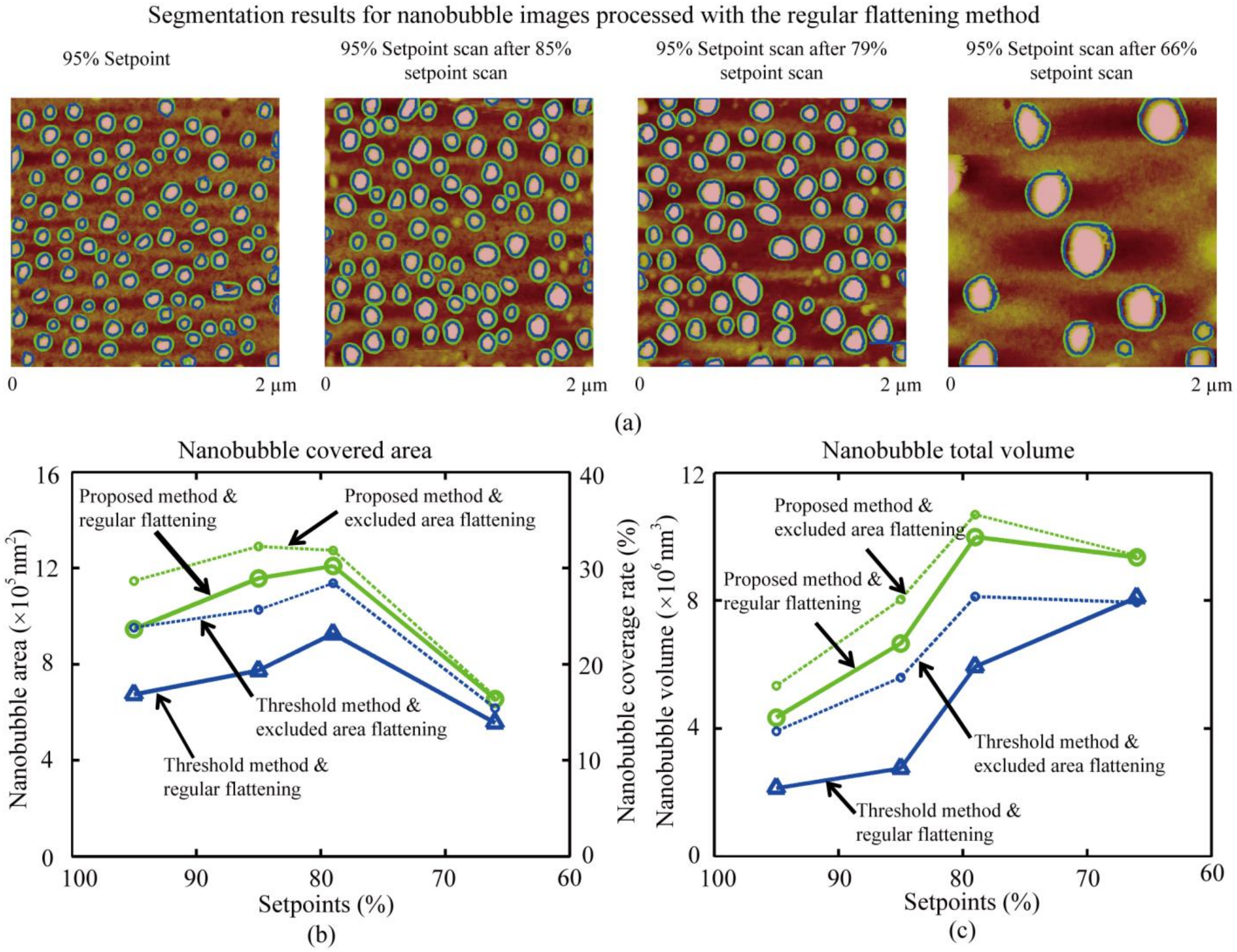

Figure S2: (a) AFM images obtained with the regular flattening method and corresponding image segmentation results. (b) Covered areas and (c) total volumes of NBs as a functions of applied setpoint. The solid lines are the result of AFM images processed with the regular flattening method, while the dotted lines are the result of AFM images processed with the excluded area flattening method.

The covered areas and volumes for all nanobubbles are shown in Figure S2(b,c), respectively. For comparison, the results for the images processed with the excluded area flattening method are also presented in Figure S2(b,c). In the figures, the dotted curves are the results for images obtained with the excluded area flattening method, while the solid curves are the results for images obtained with the regular flattening method. The blue curves are the results obtained with the threshold method, while the green curves are the results obtained with the proposed method. One can see that both the covered area and volume detected in the AFM images proposed with the excluded area flattening method are larger than that of the regular flattening method.
